# Selective interaction of PEGylated polyglutamic acid nanocapsules with cancer cells in a 3D model of a metastatic lymph node

**DOI:** 10.1186/s12951-016-0207-8

**Published:** 2016-06-23

**Authors:** Marta Alonso-Nocelo, Raquel Abellan-Pose, Anxo Vidal, Miguel Abal, Noemi Csaba, Maria Jose Alonso, Rafael Lopez-Lopez, Maria de la Fuente

**Affiliations:** Translational Medical Oncology Group, Health Research Institute of Santiago de Compostela (IDIS), Clinical University Hospital/SERGAS, Santiago de Compostela, Spain; Nanobiofar Group, Center for Research in Molecular and Chronic Diseases (CIMUS), University of Santiago de Compostela, Campus Vida, Santiago de Compostela, 15706 Spain; Cell Cycle and Oncology Group CiCLOn, IDIS, Center for Research in Molecular and Chronic Diseases (CIMUS), University of Santiago de Compostela, Campus Vida, Santiago de Compostela, 15706 Spain

**Keywords:** Nanocapsules, Metastasis, Lymph nodes, Perfusion, 3D cell culture

## Abstract

**Background:**

Metastases are the most common reason of cancer death in patients with solid tumors. Lymph nodes, once invaded by tumor cells, act as reservoirs before cancer cells spread to distant organs. To address the limited access of intravenously infused chemotherapeutics to the lymph nodes, we have developed PEGylated polyglutamic acid nanocapsules (PGA-PEG NCs), which have shown ability to reach and to accumulate in the lymphatic nodes and could therefore act as nanotransporters. Once in the lymphatics, the idea is that these nanocapsules would selectively interact with cancer cells, while avoiding non-specific interactions with immune cells and the appearance of subsequent immunotoxicity.

**Results:**

The potential of the PGA-PEG NCs, with a mean size of 100 nm and a negative zeta potential, to selectively reach metastatic cancer cells, has been explored in a novel 3D model that mimics an infiltrated lymph node. Our 3D model, a co-culture of cancer cells and lymphocytes, allows performing experiments under dynamic conditions that simulate the lymphatic flow. After perfusion of the nanocarriers, we observe a selective interaction with the tumor cells. Efficacy studies manifest the need to develop specific therapies addressed to treat metastatic cells that can be in a dormant state.

**Conclusions:**

We provide evidence of the ability of PGA-PEG NCs to selectively interact with the tumor cells in presence of lymphocytes, highlighting their potential in cancer therapeutics. We also state the importance of designing precise in vitro models that allow performing mechanistic assays, to efficiently develop and evaluate specific therapies to confront the formation of metastasis.

## Background

In many types of metastatic cancer, such as lung cancer, the regional lymph nodes act as mediators of cancer cells leading to distant organ metastases [1]. Unfortunately, the lymphatic system is not easily accessible by conventional intravenous infusion of chemotherapeutics due to their specific anatomical and physiological characteristics [2]. Therefore, eradicating cancer cells that are infiltrated in lymph nodes requires the development of specific therapeutic interventions to stop the process of metastases. In the last decades, a variety of novel chemotherapeutic agents have lead to major clinical advances, however many of them also impact immune cells, suppressing cell-mediated innate and adaptive antitumor immunity [3]. Recent data revealed that certain chemotherapeutic agents affect maturation, cytokine production and T cell stimulatory capacity of dendritic cells (DCs), which play a crucial role in the induction of antitumor immunity [4, 5]. For this reason, it is necessary to develop novel therapeutic targeted strategies to successfully improve the accessibility and selectivity of anticancer drugs to tumor cells allocated in the lymph nodes, avoiding their interaction with immune cells. Within this frame, nanotechnology offers interesting opportunities for lymphatic delivery [6–8]. At present it is known that particle size, surface charge, as well as hydrophilicity, are important parameters that can be modulated in order to enhance the ability of nanocarriers to reach the lymph nodes [9–11]. On these premises, we have developed PEGylated polyglutamic acid nanocapsules (PGA-PEG NCs) that exhibit several attractive features for an enhanced delivery of antitumor drugs to lymph node-resident metastatic cells, such as (1) small particle size (close to 100 nm), negative zeta potential and hydrophilic surface, and (2) ability to encapsulate and release anticancer drugs such as docetaxel. Considering this, and as a step further, we decided to study the efficacy and specificity of PGA-PEG NCs to interact with disseminated tumor cells in presence of immune cells. For that purpose, we focused our efforts in the development of a 3D cell culture model mimicking a metastatic lymph node that can be perfused with the proposed nanocarriers.

The use of 3D in vitro systems in drug research and development has been suggested as a potential link to bridge the gap between monolayer cultures and animal studies [12]. 3D models contribute to the design of specific assays to understand the mode of action of novel therapeutic agents [13, 14]. Different approaches have focused on the recreation of the microenvironment of the lymph nodes for a variety of applications, as for example to study the influence of the dynamic flow in structural remodeling and production of cytokines [15], to understand immunological interactions [16], or to develop artificial organs for transplantation [17, 18]. However, to the best of our knowledge, the development of a 3D model that represents the lymph nodes as metastatic entities infiltrated by tumor cells, allowing the perfusion of drugs and nanoparticles as novel therapeutic approaches, has not been addressed so far.

In this study we present the preparation and characterization of fluorescent PGA-PEG NCs, and explore their interaction with cancer cells in a specifically designed novel 3D model of a tumor-infiltrated lymph node, to determine their potential for the treatment of advanced metastatic cancer that undergoes lymphatic dissemination.

## Methods

### Preparation and characterization of DiD-loaded PGA-PEG NCs

PEGylated Polyglutamic Acid nanocapsules (PGA-PEG NCs) were prepared by the solvent displacement technique. Briefly, the organic phase, composed by 400 µl of ethanol, 200 µl of Epikuron 145 V (Cargill, Spain) dissolved in ethanol (C = 37.5 mg/ml), 100 µl of hexadecyltrimethylammonium bromide (CTAB) (Sigma-Aldrich) dissolved in ethanol (C = 15 mg/ml), 31.25 µl of Miglyol 812^®^ (Sasol Germany GmbH, Germany), and 9.5 ml of acetone, was added in aliquots of 250 µl every 15 s over an aqueous solution of methoxy-poly(ethylene glycol)-block-poly(l-glutamic acid sodium salt) (PGA-PEG Mw 35 kDa, Alamanda Polymers, USA) (C = 0.25 mg/ml in 20 ml of ultrapure water) under magnetic stirring. Then, solvents were removed in a rotary evaporator at 37 °C (Buchi Labortechnik AG, Flawil, Switzerland) up to a final volume of 10 ml. Nanocapsules were characterized by their size, polydispersity index, zeta potential, colloidal stability and morphology. Changes in physicochemical properties over time under specific conditions were measured using a Zetasizer Nano ZS^®^ (Malvern Instruments, Malvern, UK). Particle size distribution and morphology were evaluated by transmission electron microscopy (TEM) using a JEOL JEM-2010 microscope (Tokyo, Japan). DiD alone (DiD-loaded NCs) or in combination with docetaxel (Molecular Probes-Invitrogen, USA) (DiD-DCX-loaded NCs) were associated to the nanocapsules by incorporating them into the oily phase. The nanocapsules were recovered by ultracentrifugation. The concentration of DiD and docetaxel (274 μg/mL and 25 μM respectively) was quantified through UV spectrophotometry at λ = 646 nm and high-performance liquid chromatography (HPLC), respectively.

### Development and characterization of a 3D model mimicking a metastatic lymph node

A co-culture of A549 lung tumor cells and Jurkat E6.1 lymphocytes was developed in 3D scaffolds (Insert™ PS 3D Biotek LCC, North Brunswick, NJ). 50 μl of a cell suspension at a concentration of 3 × 10^5^ of each cell type (A549 cells and Jurkat E6.1 lymphocytes) was added dropwise on the surface of the scaffolds. Jurkat cells were previously labeled with Vybrant DiI cell-labeling solution (Thermo Fisher Scientific, Spain) following the manufacture’s protocol. Co-cultures were incubated for 6 h before adding 450 μl of fresh culture medium. Cells were allowed to grow for 48 h and monitored using an inverted light microscope.

Scanning electron microscopy (SEM) was performed in order to characterize the model and identify the formation of 3D cellular structures. Scaffolds were fixed in 4 % paraformaldehyde and washed extensively with phosphate buffer saline (PBS) and water. Samples were mounted on specimen holders (Ted Pella Inc., PELCO^®^) and dried at room temperature. Prior to examination, each sample was coated with gold and visualized using a Carl Zeiss Ultra Plus Scanning Electron Microscope.

Confocal Laser Scanning Microscopy (CLSM) allowed the identification of both cell populations. 3D cell co-cultures growing into Insert™ PS scaffolds were rinsed with PBS. Cell nuclei were stained with Hoechst 33342 (Invitrogen) (1:1000). Image z-stacks were captured with a 20× objective using a confocal microscope (TCS SP5, Leica Microsystems GmbH, Heidelberg), with a scanning speed of 400 Hz and image resolution of 1024 × 1024 pixels.

### Interaction of PGA-PEG NCs with cancer cells in 2D monolayers

Cells were seeded on coverslips in a multiwell-24 plate (Thermo Fisher Scientific) at 7.5 × 10^4^ cells/well in 10 % FBS supplemented medium. After 24 h, cells were incubated with dilutions of DiD-loaded and DiD-DCX-loaded PGA-PEG NCs in cell culture medium at a fluorescent probe concentration of 0.5 μg/ml (in the case of DiD-DCX-loaded PGA-PEG NCs, the concentration of docetaxel was 25 nM). After 4 h of incubation, nanocapsules were removed by PBS washing and cells were fixed with 4 % paraformaldehyde. Staining of the nuclei was performed using DAPI (Life Technologies). Samples were analyzed by CLSM at 63× magnification.

### Perfusion of PGA-PEG NCs in a 3D model of a tumor-infiltrated lymph node

Scaffolds were placed inside a bioreactor and coupled to an automatic syringe pump (New Era Pump Systems, Inc., NY, USA). DiD-loaded and DiD-DCX-loaded PGA-PEG NCs diluted in fresh culture medium (0.5 μg/ml DiD and 50 nM DCX respectively) were perfused under dynamic conditions that mimic the lymphatic flow (2 μl/min for 2 h). Scaffolds were then washed with cold PBS (10 μl/min for 1 h) and placed in fresh culture medium. After 48 h, cells were fixed with 4 % paraformaldehyde and analyzed by CLSM at 20× magnification. In a second subset of experiments, cells were recovered from the scaffolds by trypsinization and analyzed by FACS (fluorescent activated cell sorting). Also, 2D co-cultures were used as controls and collected for the analysis. Pellets were rinsed with PBS, counted, and resuspended in 500 μl of PBS–FBS 2 %. Then, tubes were placed on ice and analyzed within 2 h using a FACScan flow cytometer (BD Biosciences, Madrid, Spain), single-laser (488 nm) excitation and a LP670 filter. Results were analyzed using FlowJo Software (TreeStar Inc., Ashland, USA).

### Cell cycle analysis

Cell cycle analysis was performed in the cells recovered from the 3D co-cultures (3D). Cells from 2D co-cultures (2D) were used as controls and collected for the analysis. Cells were fixed in ice-cold ethanol, dropwise while vortexing, and then washed with PBS–FBS 2 %. Pellets were resuspended in PI (propidium iodide) staining solution and incubated for 30 min in the dark. A minimum of 10,000 events per condition was measured by flow cytometry using a FACScan flow cytometer (BD Biosciences, Madrid, Spain). The analysis of the results was performed using FlowJo Software (TreeStar Inc., Ashland, USA).

## Results

### Preparation and characterization of PGA-PEG NCs

PGA-PEG NCs, prepared by the solvent displacement technique [19, 20], exhibit interesting features that make them attractive as lymphatic drug delivery carriers (Table 1). They can be easily prepared, have a hydrophilic surface (PEG chains), and form homogeneous populations of spherical particles with a small size (116 ± 5 nm) and a negative zeta potential (−29 ± 5 mV).

**Table 1:** Physicochemical properties of PGA-PEG NCs

Physicochemical properties				EE [%]^b^		Stability^d^		Morphology^g^
PGA-PEG NCs	Size [nm]	P.I^a^	ζ Potential [mV]	DiD	DCX^c^	Storage^e^	Cell media^f^	
Blank	116 ± 5	0.1	−29 ± 5	–	–	Yes	Yes	
DiD-loaded	112 ± 12	0.1	−38 ± 2	56 ± 3	–	Yes	Yes	100 nm
DiD-DCX-loaded	109 ± 13	0.2	−39 ± 1	52 ± 1	48 ± 8	Yes	Yes	

Values are given as mean ± S.D, n = 3

^a^ *PI* polydispersity index, ^b^ *EE* encapsulation Efficiency, ^c^ *DCX* docetaxel, ^d^ Yes stands for stable nanocapsules at the conditions of study, ^e^ 4 ºC during 7 months, ^f^ 37 °C for 4 h in cell culture medium, ^g^ transmission electron microscopy micrograph

For the preparation of fluorescent nanocapsules, DiD was successfully entrapped into the oily core (final loading 0.76 % w/w with respect to the total amount of oil). The resultant DiD-loaded nanocapsules were conveniently characterized, having a mean particle size of 112 ± 12 nm, and a zeta potential of −38 ± 2 mV. In addition, fluorescent nanocapsules that co-encapsulate docetaxel (DiD- DCX-loaded PGA-PEG NCs) were successfully prepared without important changes in their physicochemical properties, having a mean size of 109 ± 13 nm and a zeta potential of −39 ± 1 mV. The encapsulation efficiency for docetaxel was 52 ± 1 (%) providing a final drug concentration in the nanoparticle suspension of 25 μM.

### Development of a 3D cell model mimicking a metastatic lymph node

**Fig. 1:**
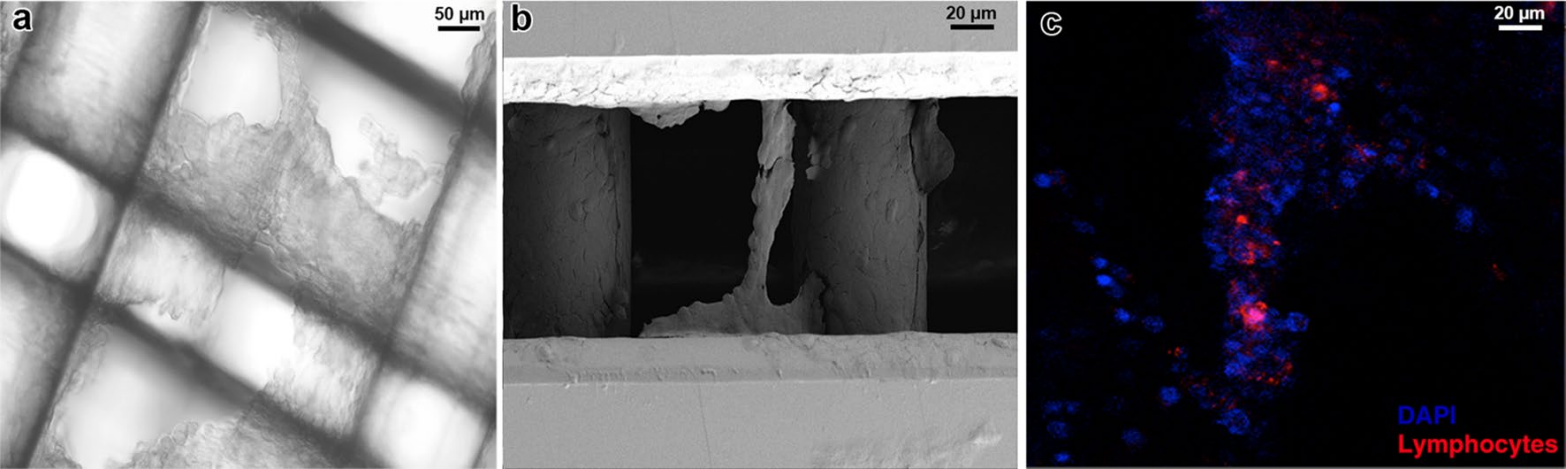
Characterization of a 3D cell model mimicking a metastatic lymph node. **a** A549 lung cancer cells and Jurkat lymphocytes were co-cultured in polystyrene scaffolds and observed as a tumor-like mass by optical microscopy. **b** Internal structure and architecture of the 3D co-culture was verified by scanning electron microscopy (SEM) at 500× magnification. **c** The presence of lymphocytes embedded in the culture (*red*: lymphocytes stained with DiI) was confirmed by confocal laser scanning microscopy (CLSM). Cell nuclei of both tumor cells and lymphocytes were counterstained with DAPI, *blue* channel

Lung tumor cells and lymphocytes were cultured together in 3D polystyrene scaffolds and allowed to grow for up to 48 h. Cell growth was directly monitored under the optical microscope (Fig. 1a). Additionally, scanning electron microscopy (Fig. 1b) provided a visual evidence of the 3D internal structure of the co-culture, showing the fibers of the scaffold covered by clusters of cells. Furthermore, using confocal microscopy we also confirmed that lymphocytes, previously stained with the stable cell tracer DiI [21], were successfully embedded in the 3D matrix (Fig. 1c, red signal).

### PGA-PEG NCs can interact with lung cancer cells and deliver their cargo

**Fig. 2:**
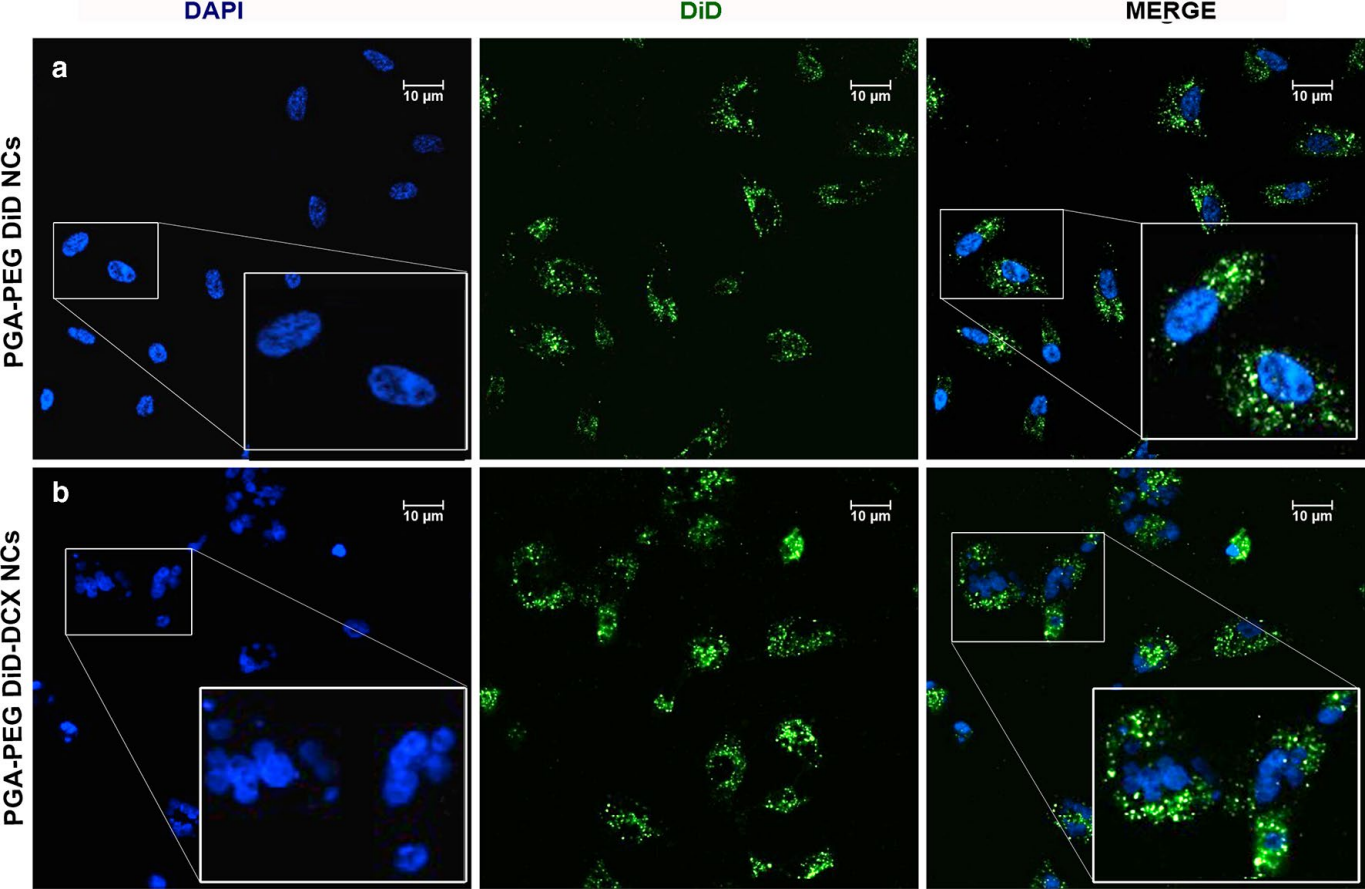
Cellular uptake studies in A549 lung adenocarcinoma cells growing in 2D monolayers. Cells were incubated for 24 h with **a** DiD-loaded and **b** DiD-DCX-loaded PGA-PEG NCs (*green* channel). Cell nuclei were counterstained with DAPI (*blue* channel). Accumulation of nanocapsules carrying DiD and docetaxel in the cells produced a cytotoxic effect characterized by nuclei fragmentation. Insets show the *boxed* areas at a higher magnification

The ability of PGA-PEG NCs to interact with lung cancer cells was first assessed in conventional cell cultures of cancer cells (2D monolayers). As observed in Fig. 2a, DiD-loaded PGA-PEG NCs were efficiently internalized by the tumor cells after 24 h of incubation, and evenly distributed into the cell cytoplasm and perinuclear area (*green* signal). On the other hand, DiD-DCX-loaded PGA-PEG NCs were similarly internalized (*green* signal) and docetaxel delivered intracellularly, leading to a cytotoxic effect characterized by cell multinucleation and enlargement (Fig. 2b).

### PGA-PEG NCs can selectively interact with lung cancer cells in the 3D co-culture cell model mimicking a metastatic lymph node

Next experiments were aimed to determine the potential of the nanocapsules to reach metastatic lung cancer cells in a selective fashion in our 3D model of a metastatic lymph node, under dynamic conditions. The perfusion flow rate of the nanocapsules was determined based on previous studies [22]. Indeed, this scenario definitely contributes to create realistic physiological barriers that hinder nanocapsules penetration into the cells. Nanocapsules perfused through the model should be able to interact with cancer cells in a short time frame (in 2D cultures cells are exposed to drugs/nanocarriers for long incubation times). Additionally, nanocapsules should penetrate into the tumor mass and have a preference for cancer cells with respect to other cells of the microenvironment, in this case immune cells.

**Fig. 3:**
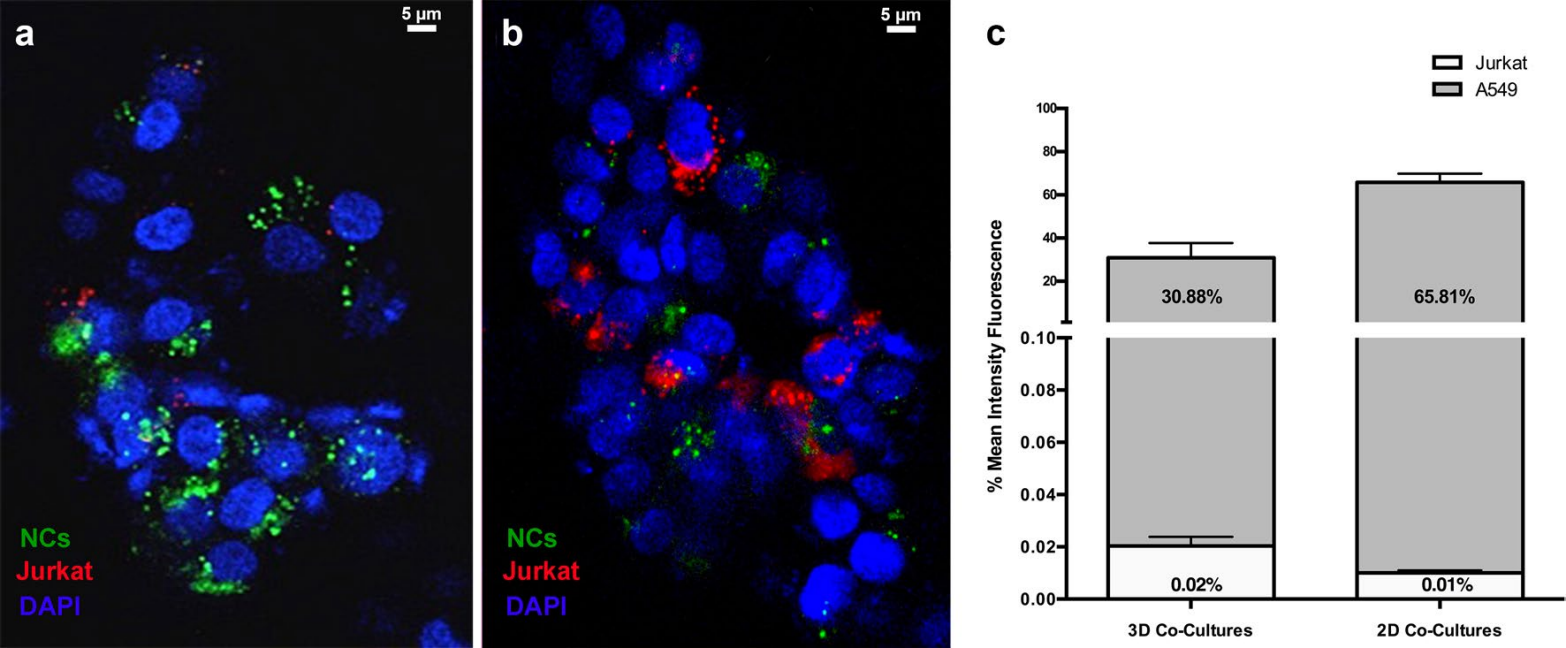
Interaction of PGA-PEG NCs with lung cancer cells in a 3D model of a metastatic lymph node. Scaffolds were perfused with DiD-loaded PGA-PEG NCs (*green* channel). **a, b** Confocal microscopy z-stacks with a different number of lymphocytes (*red* channel) showing accumulation of the fluorescent nanocapsules in cells that were negative for the DiI staining (lymphocytes, *red* channel). Cell nuclei of cancer cells and lymphocytes were counterstained with DAPI (*blue* channel). **c** *Graphical* representation of the uptake of DiD-loaded PGA-PEG NCs after perfusion in the 3D co-culture and incubation in 2D co-cultures monolayers, determined by FACS. Populations were gated separately to determine the percentage (%) of cancer cells (A549) and lymphocytes (Jurkat) that were positive for DiD (DiD-loaded PGA-PEG NCs). *Bars* represent the mean ± SD (n = 3). *NCs* nanocapsules

As observed in Fig. 3a, after perfusion of PGA-PEG NCs, fluorescence was detected around the cell nuclei in cells that were negative for DiI staining (i.e. tumor cells, as Jurkat lymphocytes were stained with DiI, red signal). Figure 3b shows a different z-stack section with a higher amount of lymphocytes, in which we can observe that green fluorescence does not co-localize with the red signal of DiI. In order to confirm these results, and to obtain quantitative data, we performed a subsequent analysis by flow cytometry shown in Fig. 3c. Both cell populations, tumor cells and lymphocytes, were recovered from the 3D co-culture and were identified separately by independent gating of each population. Data indicate that 30.9 % of the cells were positive for DiD fluorescence, the majority of which were tumor cells (30.88 %), while just 0.02 % of DiD-positive cells were identified as lymphocytes. 2D co-cultures, in which the interaction and internalization of PGA-PEG NCs takes place under static conditions, were also analyzed as controls and presented a similar fluorescent pattern. In this case, 65.82 % of the cells were positive for the DiD fluorescence. This percentage corresponded mostly to tumor cells (65.81 %) in comparison with the lymphocytes (0.01 %).

With respect to the perfusion of DiD-DCX-loaded PGA-PEG NCs, in this case and oppositely to what we observed in 2D, a cytotoxic effect was not appreciated when the cells were visualized by confocal microscopy, since cell morphology and nuclei were not affected (data not shown). Experiments to understand these differences in the activity of the nanocapsules were subsequently performed, and are disclosed in the next section.

### Cell cycle analysis

**Fig. 4:**
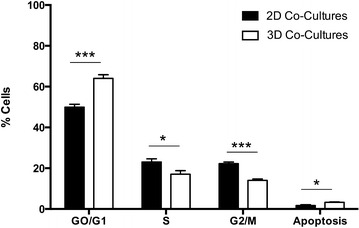
Flow cytometry analysis of the cell cycle distribution in the 3D co-culture model of a metastatic lymph node, and in 2D co-cultures monolayers. The *graph* shows the percentage of cells in each phase of the cell cycle and apoptotic cells. *Bars* represent the mean ± SD (n = 3). ***p value <0.001 *p value <0.05

Considering the mechanism of action of docetaxel, an antimitotic agent, and the absence of a cytotoxic effect in our 3D model, we decided to analyze the cell-cycle phase distribution of cancer cells (G0/G1, S and G2/M). Results show that the 3D model is characterized for the presence of a higher percentage of cells in phase G0/G1 than in G2/M, therefore suggesting a cell-cycle arrest (Fig. 4). This distribution can be attributed to the three-dimensional organization since cells recovered from the 2D co-cultures, analyzed as a control, show a significative smaller number of cancer cells under G0/G1 arrest.

## Discussion

PGA-PEG NCs, i.e. PGA-PEG coated oil in water nanoemulsions stabilized with surfactants, were successfully engineered with a mean size close to 100 nm, a round shape, a negative zeta potential and a hydrophilic surface. These characteristics are important as they are known to enhance the blood circulation time, the penetration rates into the tumor mass and the migration through the interstitial space towards the lymphatics [9, 11, 23]. Recent results show that they have an enormous potential for the treatment of metastatic lung cancer since they can efficiently reach regional lymph nodes after subcutaneous administration to mice [19]. As a step forward, in this work the objective was to elucidate the capacity of PGA-PEG NCs to efficiently interact with cancer cells, while avoiding a non-specific interaction with the immune cells.

For nanoparticle tracking, the fluorescent probe DiD was successfully entrapped into PGA-PEG NCs. Nanocapsules were stable, since they preserve their physicochemical properties during storage for up to 7 months, and in cell culture medium at 37 °C for 4 h. Additionally, DiD was not prematurely released from the formulation in the tested conditions. The gold standard treatment in lung cancer, the hydrophobic drug docetaxel, was also incorporated in the formulation with high association efficiency and reaching a concentration of 25 μM in the nanoparticle suspension. The incorporation of DiD and docetaxel did not have a significant effect on the size and surface properties of the nanocapsules, with respect to blank PGA-PEG NCs.

The capacity of PGA-PEG NCs to enter the cancer cells and release their cargo was first explored in conventional 2D cultures of lung cancer cells. As expected, results showed that PGA-PEG NCs accumulated into the cell cytoplasm and were evenly distributed in the culture. Due to their small size, nanoparticles could probably enter the cells by different mechanisms such as endocytosis and pinocytosis [24]. Moreover, the ability of PGA-PEG NCs to release the associated drug intracellularly was proved on view of the cytotoxic effect exerted after their effective internalization into lung cancer cells. However, even if the interaction of nanocarriers with cancer cells and therapeutic efficiency have been traditionally performed in 2D cell monolayers, they poorly represent the in vivo situation [25, 26].

Consequently, we aimed next to evaluate if PGA-PEG NCs could interact specifically with disseminated tumor cells in a model more representative of the metastatic situation. 3D platforms, i.e. spheroids and hydrogels, have been proposed for the evaluation of the capacity of nanoparticles to diffuse into tumors and interact with cancer cells [27, 28]. However, these models do not consider the dynamic flow. This is particularly relevant since cells in tissues are naturally subjected to fluid flow, which influence cellular functions, and crucially determines the diffusion of the nanocarriers through the tumor, limiting their contact and interaction with the cells [29, 30]. We have designed and characterized a 3D model that addresses this specific limitation and enable the perfusion of the nanocarriers under dynamic controlled conditions. An additional important feature of the 3D platform developed in this work relies on the incorporation of lymphocytes, in an attempt to reproduce the physiological environment of a metastatic lymph node. After a series of experiments intended to adjust the cell ratio and the seeding conditions, lung tumor cells and lymphocytes were successfully co-cultured in a porous 3D matrix. This complex structure composed by polystyrene and organized in four layers of polymer fibers with open interconnected pores provided cells with the appropriate support in a physiological environment, allowing exchange of nutrients and cell metabolism waste.

The results obtained after perfusion of DiD-loaded PGA-PEG NCs in the 3D model showed a selective interaction with the tumor cells. Indeed, fluorescent nanocapsules were observed around the cell nuclei of cancer cells (negative staining for DiI) under the confocal microscope. Additionally, FACS experiments allowed gathering quantitative data supporting this observation. The selective internalization of PGA-PEG NCs by cancer cells could be explained considering that biodegradable nanostructures are typically internalized by endocytosis [24] and lymphocytes are known as non-phagocytic cells [31]. Also, the hydrophilic characteristics conferred to the nanosystems by the neutral ligand poly (ethylene glycol) (PEG) might help preventing the interaction with the immune cells [32]. In addition, the internalization could have been favored by the known avidity of cancer cells for PGA as well, as previously described [33]. Due to the structure of the nanocapsules and the methodology of preparation, PGA is partially disposed onto the surface of the nanocapsules, and therefore accessible to cancer cells.

The internalization of the nanocapsules after perfusion in 3D co-cultures under dynamic conditions, was compared with that determined in 2D co-cultures after incubation under static conditions. Important differences were observed, as the percentage of positive DiD cells moved from 65 % in 2D co-cultures to 30 % in the 3D co-cultures. The reason could be that, whereas nanocapsules are delivered in a bulk solution on top of the 2D cultures, their access to the cells in 3D cultures might be hindered by architectural and physiological factors [25], as well as by a limited residence time over the cell surface. Despite of these facts, an important percentage of cells were positive after perfusion, suggesting an effective internalization process of the nanocapsules even when cells are in a dynamic 3D environment.

Regarding their potential in anticancer therapy, we confirmed that PGA-PEG NCs can efficiently entrap the hydrophobic drug docetaxel and release it intracellularly in an active form, as confirmed in conventional 2D monolayers confocal images after exposure to DiD-DCX-loaded PGA-PEG NCs, and in line with previous works in the field [34–36]. Interestingly, this antitumoral effect was not observed after perfusion of DiD-DCX-loaded PGA-PEG NCs through the 3D co-cultures. This lack of effect as compared to the significant response observed in the 2D model could be attributed to the mechanism of action of docetaxel. Indeed, docetaxel is known to bind and stabilize the microtubule network in proliferating cells thus preventing cell division [37]. However it has no effect on cells that are in the resting phase, which is the cell cycle status of cancer cells in the developed 3D model of a metastatic lymph node. In fact, when cells were cultured in 3D, the percentage of cells in phase GO/G1 was significantly increased (65 %) compared to cells cultured in 2D (30 %). This result is particularly interesting since it has been described that lymph node-resident metastatic cells often enter on a dormant state, and that the immune system has been proposed as a key player on the induction or maintenance of cell dormancy [38, 39]. Cancer dormant cells resident in the lymph nodes might eventually be re-activated and end up producing distant metastasis due to microenvironmental cues and other signals. Therefore, it seems important to deliver therapies that are specifically targeted to these dormant cells. Nanocapsules are versatile systems in which different types of drugs, from small molecular weight anticancer drugs to high molecular weight hydrophilic biomolecules (i.e. gene therapies and proteins) can be associated [19, 40, 41], and can hold potential for this particular application.

Our results highlight the importance of developing appropriate anti-cancer therapies particularly addressed to metastatic cancer cells, and in parallel, suitable in vitro models that allows understanding and validating the efficacy of the proposed therapies. Indeed, we believe that the availability of in vitro models that represent the metastatic situation could significantly contribute to the development of novel antitumoral therapies addressed to interfere cancer dissemination.

## Conclusions

We have prepared fluorescent PGA-PEG NCs with physicochemical properties that make them attractive for targeting the lymph nodes and delivery of anticancer therapeutics. We have proved a selective interaction of PGA-PEG NCs with cancer cells in presence of immune cells, and hence the potential for the treatment of cancer that undergoes lymphatic dissemination. For that, we have successfully developed a 3D model of a metastatic lymph node representative of the physiological situation, which could additionally be of utility for the screening of novel therapeutic strategies specifically addressed to tackle metastatic dormant cells.

## Abbreviations

3D: three-dimensional
PGA-PEG: PEGylated polyglutamic acid
NCs: nanocapsules
DCX: docetaxel
SEM: scanning electron microscopy
CLSM: confocal laser scanning microscopy
FACS: fluorescent activated cell sorting
